# From Theory to Practice: Translating Research into Health Outcomes

**DOI:** 10.1371/journal.pmed.0050015

**Published:** 2008-01-08

**Authors:** The PLoS Medicine Editors

## Abstract

Commenting on recent research articles which look at the potential health benefits of behaviour change, the PLoS Medicine Editors say that publication of the findings of such research is only one part of the behaviour-change process.

A research article by Kay-Tee Khaw and colleagues in this issue of *PLoS Medicine* [1] quantifies the benefits of adopting not just one but four healthy forms of behavior. Compared with people who do not engage in any of these behaviors, people in this United Kingdom study who drank in moderation, did not smoke, took exercise, and ate an adequate amount of fruit and vegetables were calculated to live an additional 14 years. Clearly, these striking findings are of relevance, not only for health researchers and public policy makers but for the entire population. If everyone in the UK adopted these four behaviors, the public health impact would be substantial. What does it take for such research findings to be turned into actual public health outcomes?

First, and most obviously, the results need to be disseminated. This is where medical and scientific journals come in, and where open-access journals, such as those published by PLoS, have such a huge advantage. With open-access journals, you don't need to be sitting in a well-funded institution to read the work; a Google search will take you straight to the paper. Second, individuals need to want to make the changes to their lifestyle that such papers recommend. Although, generally, people do want to do the best for themselves, and especially for their families, it is not always easy for the public to determine what is the “best,” given the barrage of information forced on them every week by medical journals, government reports, the popular media, and so on. And so a third key element in turning research into action is to present the information in an accessible and balanced way. If the advice is too extreme, rather than a grateful public accepting and acting on a set of recommendations, people may choose to ignore the findings. For example, when the World Cancer Research Fund released its “10 Recommendations for Cancer Prevention” [2] based on an expert report [3], media reports subsequently described a less than enthusiastic public response [4–6].

But the final essential step necessary for people to make healthy changes to their lifestyle based on research evidence has to come from society and governments providing the environment—in the widest sense—that enables such behavior change. Advice to stop smoking, for example, needs to be accompanied by an environment that encourages and enables smoking cessation. Observational studies [7] have shown that “smoke-free” legislation banning smoking in enclosed public spaces has had a clear effect on the health of bar workers in Scotland—more than any number of simple public health messages could have done. Similarly, a UK government report on obesity [8] goes beyond the simple exhortation to people to eat healthily. The report explores wider policy options aimed at addressing obesity, such as taxing obesity-promoting food, and implementing early interventions to promote better health at birth and in infancy.

Two final examples from recent studies published in *PLoS Medicine* illustrate even more clearly why individuals in isolation often cannot make the lifestyle changes they want. Bennett and colleagues [9] looked at physical activity in low-income areas in the urban United States and found evidence that people did very little walking because they feared for their safety, especially at night. But even more stark is the message that emerges from a paper by Sheri Weiser and colleagues [10] on the relationship between high-risk sexual behavior and food insecurity in Botswana and Swaziland. In Weiser's study, women who had insufficient food to feed themselves and their families were more likely to engage in high-risk sex (such as commercial sex), putting themselves at risk of HIV. As Sheri Weiser says, in an interview she gave to the Kaiser Family Foundation: “Overall what we are talking about here [are] people who had to make impossible choices. They had to make trade offs for instance…between dying now from starvation and potentially dying later from HIV.” [11]

So publication of a paper is just the first step in a complex set of processes that are needed to change behavior. We look forward to seeing whether policy makers will act upon Khaw's findings. But perhaps in the meantime it would be wise, for those of us who are able to do so, to make a few New Year's resolutions in the light of these findings.
